# Short-Term Effects of Gaseous Pollutants and Particulate Matter on Daily Hospital Admissions for Cardio-Cerebrovascular Disease in Lanzhou: Evidence from a Heavily Polluted City in China

**DOI:** 10.3390/ijerph10020462

**Published:** 2013-01-28

**Authors:** Shan Zheng, Minzhen Wang, Shigong Wang, Yan Tao, Kezheng Shang

**Affiliations:** 1 College of Atmospheric Science, Center for Meteorological Environment and Human Health, Lanzhou University, the Gansu key Laboratory of Arid Climate Change and Reducing Disaster, Lanzhou 730000, China; E-Mails: shanzhishi@163.com (S.Z.); wangminzhen1984@126.com (M.W.); shangkz@lzu.edu.cn (K.S.); 2 College of Earth and Environmental Sciences, Lanzhou University, Lanzhou 730000, China; E-Mail: taoyan@lzu.edu.cn

**Keywords:** gaseous pollutants, particle, cardio-cerebrovascular diseases, hospital admissions, time-series

## Abstract

Panel studies show a consistent association between increase in the cardiovascular hospitalizations with air pollutants in economically developed regions, but little evidence in less developed inland areas. In this study, a time-series analysis was used to examine the specific effects of major air pollutants [particulate matter less than 10 microns in diameter (PM_10_), sulfur dioxide (SO_2_), and nitrogen dioxides (NO_2_)] on daily hospital admissions for cardio-cerebrovascular diseases in Lanzhou, a heavily polluted city in China. We examined the effects of air pollutants for stratified groups by age and gender, and conducted the modifying effect of seasons on air pollutants to test the possible interaction. The significant associations were found between PM_10_, SO_2_ and NO_2_ and cardiac disease admissions, SO_2_ and NO_2_ were found to be associated with the cerebrovascular disease admissions. The elderly was associated more strongly with gaseous pollutants than younger. The modifying effect of seasons on air pollutants also existed. The significant effect of gaseous pollutants (SO_2_ and NO_2_) was found on daily hospital admissions even after adjustment for other pollutants except for SO_2_ on cardiac diseases. In a word, this study provides the evidence for the detrimental short-term health effects of urban gaseous pollutants on cardio-cerebrovascular diseases in Lanzhou.

## 1. Introduction

According to the World Health Organization (WHO), cardiovascular diseases were the leading cause of noncommunicable diseases (NCD) around the World in 2008, which accounted for 48% of all NCD deaths, or nearly 30% of all global deaths. The burden of these diseases is rising disproportionately among lower income countries and populations [1]. In China, the largest developing country in the World, it has been a main public health event in the adult population of 40 years of age and older, accounting for approximately 43% of total mortality [2]. So far, among numerous factors, it was reported that short-term exposure to ambient air pollution has been associated with an increase mortality and morbidity of cardiovascular diseases [3,4,5]. 

Previously, associations between ambient air pollution and hospital admissions for cardiovascular disease have been extensively reported in developed countries [3,6,7,8,9], and only a few studies were conducted in large Asian cities [10,11]. In Mainland China, some researchers have evaluated the adverse effect of ambient air pollution on daily mortality and morbidity in several developed large cities, including Beijing [11,12], Shanghai [13,14], Tianjin [15], and Shenyang [16]. These studies also mostly focused on the relationship between ambient air pollution and broad categories of respiratory and cardiovascular causes of death and morbidity in the more developed area of China. The association between air pollution and subgroups of cardiovascular disease of hospital admissions is quite scarce in less developed inland cities of China. Additionally, gaseous and particulate air pollution in the Lanzhou Valley of Gansu Province is a well-known public health problem, and the highest concentrations of gaseous and particulate pollutants have been documented in the urban cities in China [17], even in the World [18], less evidence is available to illustrate the effect of ambient air pollution on hospital admissions for cardiovascular disease in Lanzhou, a heavily polluted city of western China. There remains a need for replicating the findings in less developed areas, where characteristics of levels of economic development, outdoor air pollution, socio-demographic status of local residents, weather patterns and latitudes may be different [19,20].

The aim of this paper is to identify the short-term effect of major air pollutants, including sulfur dioxide (SO_2_), nitrogen dioxides (NO_2_) and particulate matter less than 10 microns in diameter (PM_10_), on hospital admissions for cardio-cerebrovascular disease in Lanzhou, western China during 2001 to 2005. We examined the associations with overall and stratified groups by gender and age. The modifying effect of season on major air pollutants was conducted to test the possible interaction. Better understanding the adverse effect of outdoor air pollution on morbidity will provide relevant information for developing public health plans and risk assessments in the ambient environment.

## 2. Materials and Methods

### 2.1. Data Collection

Lanzhou is the capital and largest city of Gansu Province in northwest China. The city has a total area of about 13,086 square kilometers (km^2^) including eight urban/suburban districts and a population of 3.14 million in the end of 2001. Our study area was limited in the urban districts of Lanzhou (1,632 km^2^) which had a population of 2.07 million by 2001. We exclude the suburban districts due to inadequate air pollution monitoring stations in that area. Lanzhou with four distinct seasons has a typical temperate, semi-arid continental monsoon climate and is characterized by dryness and coldness in winter and abundant sunlight in summer. 

Data on daily hospital admissions for cardio-cerebrovascular disease was collected between 1 January 2001 and 31 December 2005 from four largest comprehensive hospitals in Lanzhou city. Generally, over 60% of local residents mainly choose these hospitals for diagnosis and treatment of cardiovascular diseases. Relevant information for each case of daily hospital admissions were collected, *i.e.*, age, gender, living address, the date of admission and discharge, and diagnostic. The cases were coded according to the International Classification of Disease, tenth revision (ICD-10) for diseases of the cardiac diseases (ICD10:I00-I52) and cerebrovascular diseases (ICD10:I60-I69) by the clinicians in division of cardiology. We used the primary diagnoses and excluded the diseases caused by unintentional injuries or surgeries. According to the living address of cases, the patients who lived in the residential areas around the hospitals or in the urban area were chosen in this study.

**Figure 1:**
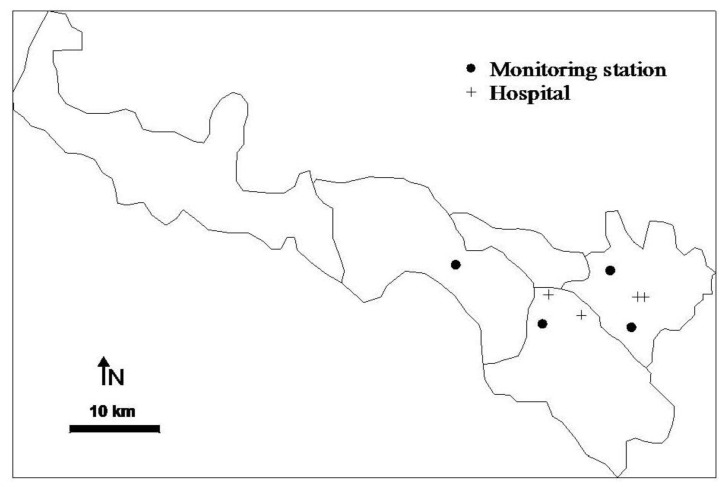
The locations of air pollutant monitoring stations and hospitals in Lanzhou.

The air pollution data during the study period, including sulfur dioxide (SO_2_), nitrogen dioxide (NO_2_), and particulate matter less than 10 μm in aerodynamic diameter (PM_10_), were obtained from the Lanzhou Environmental Monitoring Center. The center is part of a nationwide network of monitoring stations and reports daily observations to the China Environmental Monitoring Chief Station. The daily concentrations of each pollutant were averaged from the available monitoring results of four fixed stations located in the urban district of Lanzhou (Figure 1). In calculating the daily data there should be at least 75% 1-h values of that particular day, and for each monitoring station there should be at least 75% of daily data complete for the whole study period. The measurement methods for NO_2_, SO_2_, and PM_10_ were chemiluminescence, ultraviolet absorption, and tapered element oscillating microbalance, respectively. To allow adjustment for the effect of weather on hospital admissions, daily average temperature and relative humidity during the study period were obtained from the Gansu Meteorological Bureau.

### 2.2. Statistical Methods

Spearman’s correlation coefficients were used to evaluate the inter-relations between air pollutants and weather conditions. As the number of daily hospital admission data belongs to a kind of small probability event and has a Poisson distribution [21], Poisson generalized additive model (GAM) approach was used to explore the associations of major air pollutants with daily hospital admissions for cardio-cerebrovascular disease. We adjusted for day of the week (DOW) and holiday using the dummy variable. Cubic smoothing function for calendar time and year were used to control for long-term trends and seasonal patterns [22]. Other confounding factors, such as mean temperature and relative humidity were controlled by modeling with nonparametric smoothing functions in GAM model. The Akaike information criterion (AIC) was used to select the degree of freedom and measure goodness of fit [23].

Firstly, we set an independent model to explore the patterns of the relationship between the air pollutants and the hospitalizations. The independent model is described below:

Log[E(Yt|X)] = α + s(year,5) + s(time, df ) + DOW + holiday + s(tempetaturre, 3) + s(humidity, 3) + βZt(1)

where t refers to the day of the observation; E(Yt|X) denotes estimated daily hospital admissions counted on day t; α is the intercept; s( ) denotes the cubic smoothing spline; time is days of calendar time on day t; year is the year on day t; df is the degree of freedom; DOW is the day of the week on day t. β represents the log-relative rate of hospitalization associated with a unit increase of air pollutants; Zt indicates the pollutant concentrations on day t.

Secondly, we created a binary variable for season, with 0 for warm season (from May to October) and 1 for cold season (November to April). Then we added a product term between pollutant concentrations and season into the core model to test the possible interaction between air pollution and season. Model 2 as follows:

Log[E(Yt|X )] = α + β1 pollutant + β2 season + β3 pollutant × season + *COVs* (2)

where *COVs* were all time varying confounders identified in the core model (1). β_1_ signifies the main effect of the pollutant in the warm season, and (β_1_ + β_3_) was the pollutant effect in the cold season. β_2_ is a vector for coefficients of the season, and β_3_ is a vector for coefficients of the interactive term between the pollutant and the season [24].

Additionally, we examined associations stratified by gender (female and male) and age (<65 years, ≥65 years). We fitted also both single-pollutant models and multiple-pollutant models with a different combination of pollutants (up to two pollutants per model) to assess the stability of the major air pollutants effects on cardio-cerebrovascular admissions. Delayed effects were also considered to investigate with single day lags (from L0 to L3) and cumulative day lags (L01 and L03) for weather conditions and air pollutants. The current day temperature and humidity (L0) was selected to build the models due to the biggest effects. For example, a lag of 0 day (L0) represents the current-day pollutant concentrations, lag 01 means the 2-day moving average of current and previous day values. We chose the lagged day with the largest estimated effect in model (1) to analyze the other steps in the study. We also carried out sensitivity analysis to examine the impact of df selection for time trends on the effect estimates of air pollutants. The estimated effects were expressed as the increased percentage and their 95% confidence interval (95% CI) of the daily hospital admissions for cardio-cerebrovascular disease with an interquartile range (IQR) increase in daily gaseous pollutants. All analyses were running by R 2.14.0 statistical software by using mgcv package.

## 3. Results

Summary statistics of hospital admissions, air pollutant concentrations, and meteorological data are shown in Table 1. From 2001 to 2005 (1,826 days), a total of 28,243 hospital admissions for cardio-cerebrovascular disease were recorded. On average, there were approximately 10 cases/day and 6 cases/day due to cardiac diseases and cerebrovascular diseases. During the period, the average concentrations of PM_10, _SO_2 _and NO_2 _were 187.07 μg/m^3^, 79.11 μg/m^3^ and 45.81 μg/m^3^, respectively, and they were higher in the cold season than in the warm season. The average concentration of PM_10_ was higher than the national secondary ambient air quality standard in China (150 μg/m^3^). The average level of SO_2 _was higher than the national primary ambient air quality standard in China (50 μg/m^3^). The average temperature and relative humidity were 11.08 °C and 50.46%, respectively.

**Table 1:** Summary statistics of daily hospital admissions, air pollutant concentrations, and weather conditions in Lanzhou (2001–2005).

	Mean	SD	Min	P25	Median	P75	Max	IQR
Daily Hospital admissions								
Cardiac diseases	9.65	5.64	0	5	9	13	36	8
Cerebrovascular diseases	5.82	3.88	0	3	5	8	23	5
Meteorology measures								
Temperature (°C)	11.08	9.92	−12.20	2.10	11.90	20.00	30.10	17.90
Relative humidity (%)	50.46	14.03	15.90	40.20	50.70	60.30	89.80	20.1
Air pollutants concentrations								
PM_10_ (μg/m^3^)	187.07	125.78	16.00	101.00	148.00	235.00	2,561.00	134.00
SO_2_ (μg/m^3^)	79.11	61.43	2.00	37.00	58.00	106.00	371.00	69.00
NO_2 _(μg/m^3^)	45.81	29.30	4.00	25.00	37.50	56.00	260.00	31.00
Cold season ^a^								
PM_10_ (μg/m^3^)	276.04	214.39	21.00	149.00	222.00	333.00	2561.00	184.00
SO_2_ (μg/m^3^)	114.87	66.76	6.00	65.00	100.00	148.00	371.00	83.00
NO_2 _(μg/m^3^)	58.78	33.15	4.00	35.00	50.00	75.00	260.00	40.00
Warm season ^b^								
PM_10_ (μg/m^3^)	125.69	65.71	16.00	86.00	114.00	149.00	880.00	63.00
SO_2_ (μg/m^3^)	43.89	24.53	2.00	28.00	40.00	54.00	182.00	26.00
NO_2 _(μg/m^3^)	33.04	17.15	4.00	22.00	29.00	40.00	123.00	18.00

SD: standard deviation; Min: minimum; P25: ^25^th percentile; P75: ^75^th percentile; Max: maximum; IQR: inter quartile range. *^a^* Cool season: from November to April; *^b^* Warm season: from May to October.

Table 2 shows the correlations between air pollutants, temperature, and relative humidity. PM_10_, SO_2_ and NO_2_ had a strong positive correlation with each other, and were negatively correlated with temperature and relative humidity.

**Table 2:** Spearman’s correlations between daily weather conditions and air pollutant concentrations in Lanzhou (2001–2005).

	Temperature	Relative humidity	PM_10_	SO_2_
PM_10_	−0.454 ^**^	−0.320 ^**^		
SO_2_	−0.585 ^**^	−0.296 ^**^	0.624 ^**^	
NO_2_	−0.465 ^**^	−0.218 ^**^	0.643 ^**^	0.640^**^

^**^ *P* < 0.01.

Table 3 shows the estimates for the percent change in hospital admissions for cardiac and cerebrovascular disease associated with an IQR increase of air pollutants in different lag structures after adjustment for the long-term trend, DOW, holiday and weather conditions. Significant associations were found between air pollution and daily hospital admissions for cardio-cerebrovascular disease in Lanzhou. Regarding the time sequence of the associations, greater estimates were found for PM_10 _at lag 01 day (L01) in cardiac hospital admissions, SO_2_ at lag 0 day (L0) and lag 03 day (L03) in cardiac and cerebrovascular hospital admissions, while there was a more delayed relation (lag 02–03 day) for NO_2_ in cardiac and cerebrovascular disease. For instance, an IQR increase in the 2-day moving average of PM_10_ (Lag01) was associated with an increase of 2.32% (95%CI: 0.55%~4.12%) in cardiac disease admissions, An IQR increase of SO_2_ corresponded to a 2.34% (95%CI: 0.23%~4.49%) increase in the number of hospital admissions for cardiac disease at lag 0 day, and 5.53% (95%CI: 1.69%~9.53%) for cerebrovascular disease at lag 03 day. An IQR increase in the 3-day and 4-day moving average of NO_2 _(Lag02 and Lag03) was associated with an increase of 3.94% (95%CI: 1.83%~6.09%) in cardiac disease admissions, 4.76% (95%CI: 1.45%~8.19%) in cerebrovascular disease admissions.

**Table 3:** Percent change (mean and 95%CI) of the association between an IQR increase in pollutant concentrations and daily hospital admissions in Lanzhou from 2001 to 2005. *

Lag structures		Cardiac diseases		Cerebrovascular diseases	
		Change % (95%CI)	*P* value	Change % (95%CI)	*P* value
PM_10_	Single-day lag				
	0	1.28 (−0.21~2.80)	0.09	−0.77 (−3.44~1.97)	0.58
	1	1.66 (0.13~3.20)	0.03	−1.43 (−3.64~0.83)	0.21
	2	1.54 (0.06~3.05)	0.04	−1.38 (−3.47~0.76)	0.20
	3	−0.25 (−1.66~1.19)	0.74	−1.50 (−3.45~0.50)	0.14
	Cumulative-day lag				
	01	2.32 (0.55~4.12)	0.01	−1.72 (−4.60~1.25)	0.25
	02	2.13 (0.15~4.15)	0.03	−2.39 (−5.43~0.75)	0.13
	03	0.91 (−1.19~3.06)	0.40	−3.01 (−6.12~0.21)	0.18
SO_2_	Single-day lag				
	0	2.34 (0.23~4.49)	0.03	3.80 (0.63~7.08)	0.02
	1	0.81 (−1.27~2.92)	0.45	4.03 (0.87~7.29)	0.01
	2	1.82 (−0.26~3.95)	0.09	4.26 (1.07~7.55)	0.01
	3	1.24 (−0.85~3.38)	0.25	3.09 (−0.09~6.37)	0.06
	Cumulative-day lag				
	01	1.98 (−0.31~4.32)	0.09	4.71 (1.23~8.30)	0.01
	02	2.29 (−0.11~4.76)	0.06	5.40 (1.72~9.23)	<0.00
	03	2.29 (−0.21~4.86)	0.07	5.53 (1.69~9.53)	<0.00
NO_2_	Single-day lag				
	0	3.47 (1.67~5.30)	<0.00	4.06 (1.36~6.84)	<0.00
	1	2.68 (0.87~4.52)	<0.00	3.26 (0.56~6.04)	0.02
	2	2.43 (0.60~4.29)	0.01	2.43 (−0.29~5.22)	0.08
	3	1.42 (−0.39~3.27)	0.13	3.04 (0.30~5.86)	0.03
	Cumulative-day lag				
	01	3.78 (1.80~5.80)	<0.00	4.39 (1.43~7.43)	<0.00
	02	3.94 (1.83~6.09)	<0.00	4.40 (1.25~7.64)	0.01
	03	3.77 (1.55~6.03)	<0.00	4.76 (1.45~8.19)	<0.00

^* ^ Models were controlled for the time trend, DOW, holiday, mean temperature and humidity.

**Figure 2:**
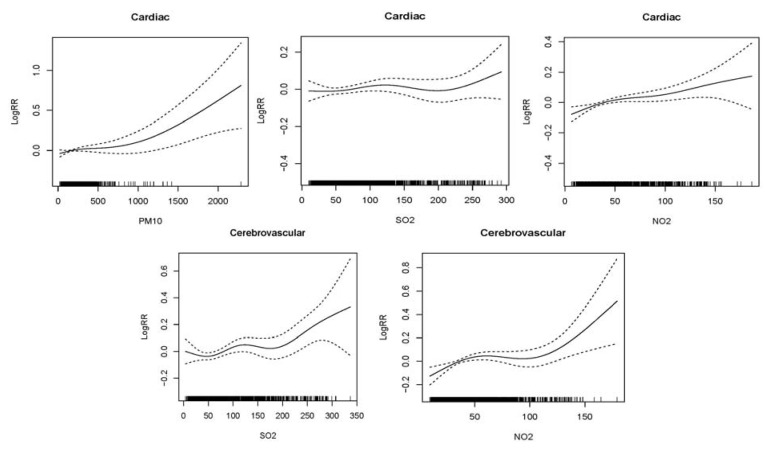
Smoothing plots of air pollutants against hospital admissions risk of cardiac and cerebrovascular diseases. X-axis is the pollutant (PM_10 _or SO_2_ or NO_2_) (µg/m^3^). The solid lines indicate the estimated mean percentage of change in daily hospital admission, and the dotted lines represent twice the point-wise standard error ^*^. ^*^ The greatest effects of single-day lag 0 (L0) SO_2_ and cumulative-day lag (Lag01 for PM_10_, lag02 for NO_2_ ) were used for cardiac hospital admissions; cumulative-day lag 03 (L03) SO_2_ and NO_2_ were used for cerebrovascular hospital admissions. All models were controlled for the time trend, DOW, holiday, mean temperature and humidity.

Figure 2 shows graphically the exposure-response relationships between air pollutants and daily hospital admissions for cardio-cerebrovascular disease in the single-pollutant models. There were similar positive linear relationships between air pollutants and cardiac and cerebrovascular hospital admissions, which indicated the relative risk of hospital admissions increased as air pollution increased in Lanzhou during the study period. Since there was no significant association between cerebrovascular disease and PM_10_, we did not give the figure.

Table 4 shows the magnitude of the effects of air pollutants varied with gender and age. The significant associations were found between air pollutants and hospital admissions by gender. The effects of gaseous pollutants showed the greater association with cardiac-cerebrovascular diseases than the particulate matter in male and female. Percent increases of cardiac-cerebrovascular diseases in female were significantly greater than in male except for SO_2_ on cerebrovascular diseases. In two different age groups, the significant effects of air pollutants on hospital admissions clearly increased with age with the exception of the effect of SO_2_ on cardiac hospital admissions.

**Table 4:** A Percent change (mean and 95%CI) of daily hospital admissions with an IQR increase in pollutant concentrations by gender and age group in Lanzhou from 2001 to 2005 *.

		Cardiac diseases			Cerebrovascular diseases		
		n ^a^	Change % (95%CI)	*p* value	N ^a^	Change % (95%CI)	*p* value
Gender							
Male	PM_10_	5.94	2.05 (−0.17~4.32)	0.07	3.79	−0.21 (−3.47~3.17)	0.90
	SO_2_		2.33 (−0.72~5.49)	0.14		6.47 (2.41~10.68)	<0.00
	NO_2_		3.64 (0.54~6.84)	0.02		4.45 (0.38~8.68)	0.03
Female	PM_10_	3.71	2.99 (0.10~5.96)	0.04	2.03	−3.72 (−8.39~1.19)	0.14
	SO_2_		5.82 (1.63~10.17)	0.01		2.65 (−3.53~9.24)	0.41
	NO_2_		6.25 (1.98~10.70)	<0.00		6.06 (0.47~11.95)	0.03
Age							
<65	PM_10_	4.15	2.19 (−0.35~4.80)	0.09	2.98	−0.65 (−4.39~3.25)	0.74
	SO_2_		2.82 (−0.41~6.16)	0.09		3.97 (−1.32~9.55)	0.14
	NO_2_		3.04 (−0.15~6.33)	0.06		2.92 (−1.44~7.47)	0.19
≥65	PM_10_	5.50	2.47 (0.03~4.98)	0.04	2.84	−1.98 (−5.79~1.98)	0.32
	SO_2_		2.05 (−0.69~4.86)	0.14		6.15 (0.77~11.83)	0.02
	NO_2_		4.60 (1.83~7.45)	<0.00		5.85 (1.15~10.78)	0.01

* The greatest effects of single-day lag 0 (L0) SO_2_ and cumulative-day lag (Lag01 for PM_10_, lag02 for NO_2_) were used for cardiac hospital admissions; single-day lag 0 (L0) PM_10_ and cumulative-day lag 03 (L03) SO_2_ and NO_2_ were used for cerebrovascular hospital admissions. All models were controlled for the time trend, DOW, holiday, mean temperature and humidity.^ a ^Number of daily hospital admissions.

Table 5 shows the effects of air pollutants on daily hospital admissions across seasons, with the interaction terms of pollution concentrations and the season. We observed the significant associations both in the cold season and warm season. In the cold season, the significant positive relationships were observed between PM_10_ and NO_2_ and cardiac disease hospitalizations. Similarly, significant associations of NO_2 _and SO_2_ with cerebrovascular disease hospitalizations were observed. In the warm season, only NO_2 _was significantly associated with increased cardiac disease admissions, and the effect estimates were higher than that in the cold season.

**Table 5:** Percent change (mean and 95%CI) of daily hospital admissions associated with an IQR increase in pollutant concentrations modified by season level in Lanzhou from 2001 to 2005 *.

Season ^a^	Cardiac diseases			Cerebrovascular diseases		
	n ^b^	Change % (95%CI)	*p* value	n ^b^	Change % (95%CI)	*p* value
Cold season						
PM_10_	9.72	7.92 (1.91~14.30)	0.01	5.58	−4.67 (−13.0~4.58)	0.31
SO_2_		2.02 (−0.21~4.29)	0.08		5.76 (1.65~10.04)	0.01
NO_2_		2.94 (0.68~5.25)	0.01		4.58 (1.02~8.27)	0.01
Warm season						
PM_10_	9.59	5.81 (−0.20~12.18)	0.06	6.06	−2.90 (−6.11~0.41)	0.08
SO_2_		1.97 (−3.82~8.10)	0.51		2.81 (−7.84~14.68)	0.62
NO_2_		8.41 (3.69~13.34)	<0.00		5.39 (−1.74~13.03)	0.14

* The greatest effects of single-day lag 0 (L0) SO_2_ and cumulative-day lag (Lag01 for PM_10_, lag02 for NO_2_) were used for cardiac hospital admissions; single-day lag 0 (L0) PM_10_ and cumulative-day lag 03 (L03) SO_2_ and NO_2_ were used for cerebrovascular hospital admissions. All models were controlled for the time trend, DOW, holiday, mean temperature and humidity. ^a.^ Product term of a pollutant and season (binary variables for the cold or warm season) was added to the model. ^b ^Number of daily hospital admissions.

**Table 6:** Percent change (mean and 95%CI) of daily hospital admissions of Lanzhou associated with an IQR increase in pollutant concentrations in single and multiple pollutants models. *

Models	Cardiac diseases		Cerebrovascular diseases	
	Change % (95%CI)	*p* value	Change % (95%CI)	*p* value
PM_10_	2.13 (0.15~4.15)	0.03	−2.39 (−5.43~0.75)	0.13
+ SO_2_	1.97 (−0.03~4.01)	0.05	−1.84 (−4.88~1.29)	0.25
+ NO_2_	1.27 (−0.80~3.38)	0.23	−2.10 (−5.14~1.05)	0.19
+ SO_2_+ NO_2_	1.27 (−0.80~3.38)	0.23	−2.04 (−5.10~1.12)	0.20
SO_2_	2.29 (−0.11~4.76)	0.06	5.40 (1.72~9.23)	<0.00
+ PM_10_	2.58 (−0.29~5.54)	0.08	8.52 (4.65~12.53)	<0.00
+ NO_2_	−0.24 (−3.57~3.22)	0.89	5.82 (1.34~10.50)	0.01
+ PM_10_+ NO_2_	−0.05 (−3.45~3.46)	0.98	5.86 (1.37~10.54)	0.01
NO_2_	3.94 (1.83~6.09)	<0.00	4.40 (1.25~7.64)	0.01
+ PM_10_	4.16 (1.55~6.84)	<0.00	6.97 (3.59~10.46)	<0.00
+ SO_2_	4.71 (1.64~7.88)	<0.00	4.00 (0.08~8.07)	0.04
+ PM_10_+ SO_2_	4.18 (1.02~7.45)	0.01	4.82 (0.81~8.98)	0.02

* Cumulative-day lag 02 (L02) of air pollutants was used for cardiac and cerebrovascular hospital admissions. All models were controlled for the time trend, DOW, holiday, mean temperature and humidity.

Table 6 compares the results of single-pollutant and multiple-pollutant models. For cardiac disease hospital admissions, the effect of PM_10_ reduced and became insignificant after adjusting other pollutants, SO_2_ has no significant effect either before or after adjustment for co-pollutants. The effect of NO_2_ increased and remained significant after adding the other pollutants. As for cerebrovascular diseases hospital admissions, PM_10_ was not found significant in both single and multi-pollutant models. The effect of SO_2_ did not alter much by adding NO_2__, _or both NO_2_ andPM_10_, but its effect became larger after adding PM_10_; NO_2_ results were similar with SO_2_.

## 4. Conclusions

In our study, we found a significant association between ambient air pollutants and the daily hospital admissions for cardio-cerebrovascular disease in Lanzhou during 2001–2005. The effects of SO_2 _and NO_2_ were associated with the increased number of cardiac and cerebrovascular disease admissions. The people aged 65 years and older was associated more strongly with gaseous pollutants than the younger. Although more significant associations were found in the cold season, the effect of NO_2_ was apparent in the warm season. These findings may give suggests for the local government to take steps to protect human health in Lanzhou.

Some previous studies have been conducted to estimate the effects of particulates on cardiovascular diseases in mortality [5,20,25,26,27] and morbidity [4,7,21,28,29] in many regions of the world. We found a significant impact of PM_10_ on hospital admissions for cardiac disease, but no such pattern was found between PM_10_ and cerebrovascular diseases. Our findings confirm those of earlier large analyses in European [3,30], Australian and New Zealand [8] cities. Although some significant results of several studies have showed the association between PM_10_ and stroke [31,32], little evidence clearly points to a possible effect on stroke, the biologic mechanisms for these associations also have not been fully established well. The particulates pollution was mainly caused by fossil-fuel combustion, local heavy industry emission and remote transport of dust storm in Lanzhou. Recent biological studies [33,34,35] provided the evidences that PM_10_ can influence important circulatory parameters including fibrinogen levels, counts of platelets and white or red blood cells that were increased risk factors of cardiovascular disease. Additional studies with more detailed data obtained from a wide variety of cities throughout the world are needed to confirm a major argument in favor of causality. 

SO_2_ is a gaseous pollutant mainly emitted by fuel combustion and has been found to be significantly associated with the increased cardiovascular [6,36], ischemic heart disease [6], hypertension [37], heart failure [36], and stroke [32] disease admissions. Findings of our study also showed the significant associations between SO_2_ and cardiac and cerebrovascular disease admissions, but some researches [4,36] found inconsistent results which might be due to correlations with PM_10_ and NO_2_. Although SO_2 _didn’t show a very robust effect on cardiac diseases when adding NO_2_ or both NO_2_ and PM_10_ in multiply pollutant models, a recent study [6] in Europe provided the evidence that SO_2_ pollution may play an independent role in triggering ischemic cardiac events, which indicate the adverse effect of SO_2_ on human health as an independent air pollutant. The potential biological mechanisms have been proposed for the relationship. As the SO_2_ inhalation concentration rise, the osmotic fragility ratios, and methemoglobin and sulfhemoglobin values were significantly higher in blood [38]. Recent studies also found that SO_2 _had relevant effects on blood pressure, heart rate variability [39] and ventricular arrhythmia [40]. 

Of the pollutants we considered, the significant associations with NO_2_ were the most robust in single and multiple pollutant models in our study. This result was consistent with some studies [32,37], but conflict with the other findings [4]. According to the research [41], motor vehicle exhaust and industrial emissions were the major anthropogenic sources of NO_2_ in Lanzhou. The adverse effect of NO_2_ on cardiovascular diseases has been widely reported mainly in urban areas. Studies on the biological mechanism of cardiovascular impairments due to gaseous pollutants found that the increase in NO_2_ was associated with increased plasma fibrinogen [42], platelet counts [33] and arrhythmia [43]. Increasing NO_2_ exposure also found to be associated with decreasing the standard deviation of all normal-to-normal intervals (SDNN) [44] which was a risk marker in patients with left ventricular systolic dysfunction (LVSD) [45]. All these adverse effects may in turn activate circulatory pathways and impair cardiovascular function. 

Elderly people had higher estimates for cardiac-cerebrovascular admissions for gaseous pollutants than the younger except the effect estimate of SO_2_ on cardiac hospital admissions in this study. Some studies have suggested that gaseous pollutant from outdoor sources has stronger effect on the elderly than younger people [8,36]. Another research of the public health and air pollution in Asia (PAPA) study [46] showed sex, age and education may modify the health effects of outdoor air pollution in Shanghai, and females and the elderly were more vulnerable to outdoor air pollution. Actually elderly people constitute the largest proportion of cardiovascular illness and death; they were usually susceptible to air pollution as the high-risk group compared with younger people. Especially for those patients with cardiorespiratory diseases, we hypothesized that these elderly people may not adjust well to the serious air pollution. So the identification of target diseases and high risk groups would be useful in finding suitable air quality guidelines in environmental health [36].

Our findings showed the significant association between air pollutants and hospital admissions in both the cold and warm season. Generally, the concentrations of air pollutants were higher and more variable in the cold season than in the warm season in Lanzhou, and the more significant effects of air pollutants on cardio-cerebrovascular disease admissions were found accordingly in the cold season, which is consistent with several studies in Shanghai [14] and Hong Kong [4]. The observation of stronger effects of air pollutants in the cold season might be due to the special topography characteristics and regional meteorology in Lanzhou. Lanzhou is located in a narrow (2–8 km width) and long (40 km), valley surrounded by mountains with the Yellow River flowing across the city. And in such a valley, the weather is generally stable with weak wind and strong inversions. The topographic characteristics make Lanzhou vulnerable to the air pollution, particularly in winter [47]. Additionally, except the traffic and industrial process emissions, increased winter domestic coal consumption exerted a strong influence on ambient pollutant concentrations [41]. On the other hand, we also observed the high effect of NO_2_ in the warm season. The consistent results were also found that the stronger effect of NO_2_ on cardiovascular disease at the higher temperature level in Wuhan [48] and Taiwan [32]. There were some possible reasons for this phenomenon. Firstly, with the development of urbanization process, the volumes of vehicular traffic were increasing which might be the main resource of NOx. In addition, in the warm season, with higher temperature and insolation rates, ambient ozone levels increased which may result in the oxidation processes of NO to NO_2_ enhanced [41]. So, it was considered the interaction effect of gaseous pollutants exposure and the season may be existed and the potential mechanism should be studied further.

Our study has several limitations. Firstly, like other ecological studies, we just use the average of monitoring results across different stations as surrogates of personal exposure level to ambient air pollution. The use of ambient rather than personal exposure measures is expected to result in exposure misclassification. However, this misclassification is expected to lead one to underestimate the relative risk [49]. And the difference between these proxy values and the true exposures are an inherent and unavoidable type of measurement error [14]. Secondly, due to the date of symptom onset likely preceded the date of admission in a proportion of cases, there is a delay from the onset of an increase in the air pollutant level to hospital admission for cardio-cerebrovascular diseases. Thirdly, the data of hospital admissions we collected was limited in four hospitals, a select basis may exist in our study.

In summary, we observed significant associations between exposure to gaseous pollutants (SO_2_ and NO_2_) and increased hospital admissions for cardio-cerebrovascular disease in Lanzhou. Our finding strengthens the evidence of the short-term effect of gaseous pollutants on cardio-cerebrovascular diseases for morbidity in a heavily polluted city of western China. This work may have many implications for the redesigning of public health policy regarding the air pollution in Lanzhou.
